# *Vibrio parahaemolyticus-*specific *Halobacteriovorax* From Seawater of a Mussel Harvesting Area in the Adriatic Sea: Abundance, Diversity, Efficiency and Relationship With the Prey Natural Level

**DOI:** 10.3389/fmicb.2020.01575

**Published:** 2020-07-08

**Authors:** Donatella Ottaviani, Silvia Pieralisi, Elena Rocchegiani, Mario Latini, Francesca Leoni, Francesco Mosca, Alberto Pallavicini, Pietro Giorgio Tiscar, Gabriele Angelico

**Affiliations:** ^1^Laboratorio Controllo Alimenti, Istituto Zooprofilattico Sperimentale dell’Umbria e delle Marche, Ancona, Italy; ^2^Facoltà di Medicina Veterinaria, Università degli Studi di Teramo, Teramo, Italy; ^3^Facoltà di Biologia, Università degli Studi di Trieste, Trieste, Italy; ^4^Istituto Nazionale di Oceanografia e di Geofisica Sperimentale, Trieste, Italy

**Keywords:** *V. parahaemolyticus*-specific *Halobacteriovorax*, *Vibrio* spp., *V. parahaemolyticus*, *V. cholerae* non-O1/O139, Adriatic Sea

## Abstract

This research aimed to study the abundance and molecular diversity of *Vibrio parahaemolyticus*-specific *Halobacteriovorax* strains isolated from seawater of the Adriatic Sea and the relationship between predator and prey abundances. Moreover, predator efficiency of the *Halobacteriovorax* isolates toward *V. parahaemolyticus* and *Vibrio cholerae* non-O1/O139 strains was tested. *V. parahaemolyticus* NCTC 10885 was used as primary host for the isolation of *Halobacteriovorax* from seawater by the plaque assay. Molecular identification was performed by PCR detection of a fragment of the 16S rRNA gene of the *Halobacteriovoraceae* family members. Moreover, 700 bp PCR products were sequenced and compared between them and to clones described for other sampling sites. *Vibrio* counts were performed on TCBS agar from 100 ml of filtered water samples and presumptive colonies were confirmed by standard methods. Predatory efficiency of *Halobacteriovorax* isolates was tested by monitoring abilities of 3-day enrichments to form clear lytic halos on a lawn of *Vibrio* preys, by the plaque assay. Out of 12 seawater samples monthly collected from June 2017 to May 2018, 10 were positive for *V. parahaemolyticus* specific *Halobacteriovorax* with counts ranging from 4 to 1.4 × 10^3^ PFU per 7.5 ml. No significant relationship was found between *Halobacteriovorax* and *Vibrio* abundances. The 16SrRNA sequences of our *Halobacteriovorax* strains, one for each positive sample, were divided into three lineages. Within the lineages, some sequences had 100% similarity. Sequence similarity between lineages was always <94.5% suggesting that they may therefore well belong to three different species. All *Halobacteriovorax* isolates had the ability to prey all tested *Vibrio* strains. Additional research is necessary to assess whether stable strains of *Halobacteriovorax* are present in the Adriatic Sea and to understand the mechanisms by which *Halobacteriovorax* may modulate the abundance of *V. parahaemolyticus* and other vibrios in a complex marine ecosystem.

## Introduction

In many ecological communities, predation has a key role in regulating community structure or function. In the marine environment, predation has been extensively explored in animals, microbial eukaryotes and viruses (Baum and Worm, 2009) while predation by bacteria is less well understood (Williams et al., 2015). The genus *Halobacteriovorax*, in the class Deltaproteobacteria, family Bacteriovoracaceae, consists of small, Gram-negative, flagellated, marine predator bacteria that are members of a broader group of predatory bacteria known as *Bdellovibrio* and like organisms (BALOs), also including non-marine (terrestrial and freshwater) forms (Williams and Baer, 2005). BALOs enter into a susceptible Gram-negative prey bacterium and reside within the periplasmic space where they use the cytoplasmic nutrients of the prey to support growth and replication. The replicative form, known as bdelloplast, extends within the prey and divides into progeny cells that are released as soon as the host is lysed and are able to attack other preys (Williams and Baer, 2005). The genus *Halobacteriovorax*, according to the results of the analysis of the 16S rRNA gene sequence, includes two species *Halobacillus litoralis* and *Halobacteriovorax marinus* for which the similarity between the type strains is 92.61% (Koval et al., 2015). Recently, the new species *Halobacteriovorax vibrionivorans* has been also proposed (Ye et al., 2019). Furthermore, analysis of the 16S rRNA gene sequence from *Halobacteriovorax* saltwater strains identified multiple distinct phylogenetic clusters in different marine habitats, grouped based on a sequence identity with values >96.5% (Pineiro et al., 2007). Many of these *Halobacteriovorax* could represent potential new species. *Halobacteriovorax* favors predation on *Vibrio* species and other saltwater prey (Rice et al., 1998; Chen et al., 2012; Koval et al., 2015; Ye et al., 2019). Different lineages within this genus favor estuarine or marine waters and some isolates have been found in salt lakes (Koval et al., 2015). There are no known freshwater isolates (Koval et al., 2015). Recent studies reported that *Halobacteriovorax* were capable of containing *Vibrio parahaemolyticus* levels in seawater and oysters, at laboratory scale (Chen et al., 2011; Li et al., 2011; Richards et al., 2012, 2016; Williams et al., 2015; Ottaviani et al., 2018a). Furthermore, there is evidence that *Halobacteriovorax* contributes to *Vibrio vulnificus* mortality in a simulated natural seawater system with greater efficiency than other natural predators, such as bacteriophages (Chen et al., 2018). A previous study demonstrated that *Halobacteriovorax* was part of the coral microbiome (Welsh et al., 2016). In light of this evidence to understand if *Halobacteriovorax* has a role in modulating the natural levels of *V. parahaemolyticus* and other vibrios in the marine environment it is essential to know its natural abundance and understand in details how the predator infects and kills the prey. Quantitative PCR has been used to detect total *Halobacteriovorax* abundances in the marine environment, but it does not identify strains capable of targeting specific host bacteria (Zheng et al., 2008). On the other hand, Pineiro et al. (2007) isolated and characterized selected *Halobacteriovorax* phylotypes against *V. parahaemolyticus* in seawater, but results were not quantitative. To date, only two previous (plaque assay-based) studies have documented the abundance of *V. parahaemolyticus* specific *Halobacteriovorax* in seawater (Richards et al., 2013; Ottaviani et al., 2018a). However, as far as we know, there are no data on correlations between levels of *Halobacteriovorax*, *V. parahaemolyticus* and total vibrios in the marine environment. *V. parahaemolyticus* strains producing thermostable direct haemolysin (TDH) and/or TDH-related haemolysin (TRH) are recognized as a cause of diarrhoeal diseases worldwide, with bivalves, eaten raw or undercooked being the most frequent sources of infection (Potasman et al., 2002; Letchumanan et al., 2014). In Italy *Halobacteriovorax* and pathogenic vibrios, including *V. parahaemolyticus* and non-O1/O139 *Vibrio cholerae*, have been isolated in seawater and mussels coming from the Adriatic Sea (Masini et al., 2007; Ottaviani et al., 2018a). Moreover, in the last years, illness due to *V. parahaemolyticus* and non-O1/O139 *V. cholerae*, with mussels or seawater of Adriatic Sea as the source of infection, has been reported (Ottaviani et al., 2010, 2013, 2018b). In this work we studied the abundance and molecular diversity of *V. parahaemolyticus*-specific *Halobacteriovorax* strains isolated from seawater of a mussels growing area at the Conero Riviera of the Adriatic Sea. From the same samples *V. parahaemolyticus* and total vibrios were also counted and the relationship between these and *Halobacteriovorax* was studied. Finally, the predatory efficiency of *Halobacteriovorax* isolates toward *V. parahaemolyticus* and *V. cholerae* non-O1/O139 strains from clinical and environmental sources, the majority linked to the Adriatic sea of Italy, was tested.

## Materials and Methods

### Sampling Site

**FIGURE 1:**
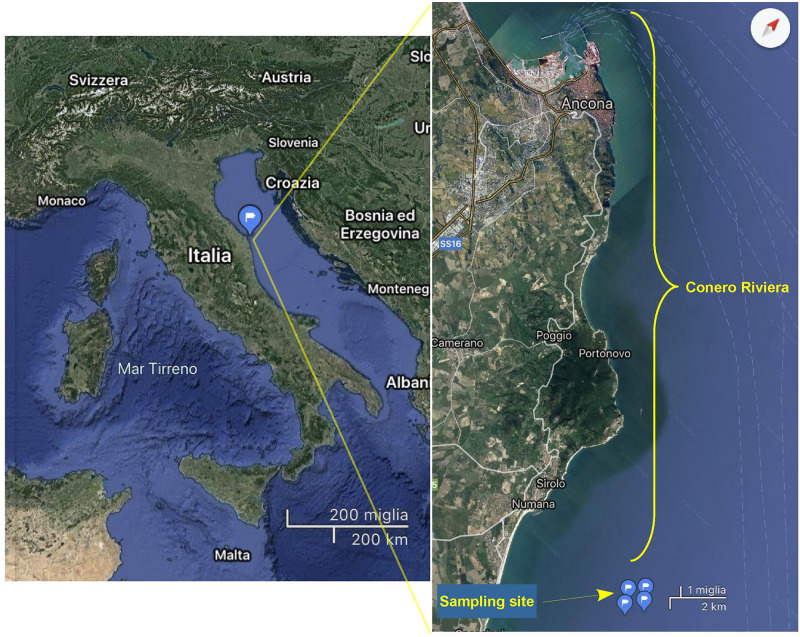
Map of the Conero Riviera showing the location of the sampling site.

The Conero Riviera, located halfway of the Italian Adriatic coast, is a coastal marine ecosystem with an extension of 20 km and a low anthropic impact. This riviera includes several important sea towns, such as Ancona, Portonovo, Sirolo, and Numana, and lies in a protected area representing the most important marine reserve of the Marches where there are also many natural banks of mussels (*Mytilus galloprovincialis*) (Figure 1). Subsurface seawater samples were monthly collected from a site of approved mussels growing area in the Conero Riviera (43°30′12″N–13°41′12″E) from June 2017 to May 2018 (Figure 1). Two liters of seawater for each sample were collected using sterile polypropylene bottles. Water temperature and salinity were determined in situ with a multiparametric probe (Handy Gamma, Oxyguard, Denmark). All samples were transported to the laboratory on ice for analysis. Salinity remained steady between 38 and 39 ppt. Seawater was immediately transported to the laboratory in an insulated cooler and analyzed within 4 h. The same seawater samples that were used for *Halobacteriovorax* analyses were also used for *V. parahaemolyticus* and total *Vibrio* analyses.

### Prey Strains

*Vibrio parahaemolyticus* NCTC 10885 strain was used as prey. Prey specificity and predatory efficiency of *Halobacteriovorax* isolates were tested on *V. parahaemolyticus* and *V. cholerae* strains of environmental and clinical origin, the majority directly or indirectly linked to the Adriatic sea of Italy (Table 1). For all *Vibrio* strains, fresh enrichments were prepared from a stock culture grown on 3% NaCl Luria-Bertani broth until prey reached an OD_600_ of 0.20 (∼1.8 × 10^8^ CFU/ml).

**TABLE 1:** *Vibrio* preys tested with *Halobacteriovorax* strains.

No.	Prey strains Toxin genes*	Source and year of isolation	Origin
1	*V. parahaemolyticus tdh* + **; trh−***; *toxRS* + (pandemic strain)	Feces, 2007	Mussels of Adriatic sea, Italy as the most probable source of infection
2	*V. parahaemolyticus tdh*−*/trh*−; *toxRS*−	Feces, 2010	Mussels of Adriatic sea, Italy as the most probable source of infection
3	*V. parahaemolyticus tdh*−; *trh*−; *toxRS*−	Feces, 2010	Mussels of Adriatic sea, Italy as the most probable source of infection
4	*V. parahaemolyticus tdh*−*/trh* +; *toxRS*−	Marine water, 2011	Conero Riviera, Italy
5	*V. parahaemolyticus tdh* + */trh*−; *toxRS*−	Mussels, 2014	Conero Riviera, Italy
6	*V. parahaemolyticus tdh*−; *trh*−; *toxRS*−	Mussels, 2015	Conero Riviera, Italy
7	*V. parahaemolyticus tdh*−; *trh* +; *toxRS*	Mussels, 2016	Conero Riviera, Italy
8	Non O1/O139 *V. cholerae hly*AClass−; *hly*ET+; *ctx*A−; *tcp*AClass+; *tcpA*ET−; *stn/sto*−	Subcutaneous tissue, 2009	Seawater of Adriatic sea, Italy, as the most probable source of infection
9	Non O1/O139 *V. cholerae hly*AClass−; *hly*ET+; *ctx*A−; *tcp*AClass−; *tcpA*ET−; *stn/sto*−	Subcutaneous tissue, 2012	Seawater of Adriatic sea, Croazia, as the most probable source of infection
10	Non O1/O139 *V. cholerae hly*AClass−; *hly*ET+; *ctx*A−; *tcp*AClass−; *tcpA*ET−; *stn/sto* +	Marine water, 2011	Conero Riviera, Italy
11	Non O1/O139 *V. cholerae hly*AClass−; *hly*ET+; *ctx*A−; *tcp*AClass−; *tcpA*ET−; *stn/sto*−	Feces, 2012	Bivalves as the most probable source of infection
12	Non O1/O139 *V. cholerae hly*AClass−; *hly*ET+; *ctx*A−; *tcp*AClass−; *tcpA*ET−; *stn/sto*−	Mussels, 2014	Conero Riviera, Italy
13	*V. parahaemolyticus* NCTC 10903		
14	*V. parahaemolyticus* NCTC 10884		
15	*V. cholerae O1* NCTC 9459		

* tdh: thermostable direct haemolysin gene; *trh*: TDH-related haemolysin gene; *tox*RS: pandemic genetic marker; *ctx*A: cholera toxin gene; *stn/sto*: heat stable enterotoxin gene; *hly*AClass: hemolysin classical gene; *hly*ET: hemolysin El Tor gene; *tcp*AClass: toxin-coregulated pilus classical gene; *tcp*AET: toxin-coregulated pilus El Tor gene; ** +: positive; ***−: negative.

### Halobacteriovorax Enumeration

It was performed by a double layer agar plating technique (Richards et al., 2012). The prey was grown in LB–3% NaCl broth until it reached an OD_600_ of 0.20 (∼1.8 × 10^8^ CFU/ml). For the analysis, 500 ml of test seawater was first filtered through a 0.45-μm, 500-ml filter to remove particulates and bacteria. Then, the filtered seawater was serially diluted 10-fold in sterilized artificial seawater (30 ppt). For each assay, 25 ml of bottom-layer Pp 20 agar (polypeptone peptone supplemented with Bacto agar) were dispensed into a 100-mm Petri dish and allowed to harden. Top agar was pipetted into sterile glass tubes (7.5 ml/tube) while the agar was hot and allowed to cool to 48°C in a water bath. The plaque assay was conducted by combining 1 ml of host *V. parahaemolyticus* culture, at an OD_600_ of 0.20 and 7.5 ml of undiluted and diluted test seawater to 7.5 ml of molten (48°C) Pp 20 agar in tubes. The tubes were inverted 3 times to mix and poured on top of the existing bottom layer. Counts of *Halobacteriovorax* were performed after incubation at 26°C for 7 days. The number of viable *Halobacteriovorax* isolates was estimated as PFU per 7.5 ml seawater.

### *Halobacteriovorax* Molecular Identification and Sequencing Analysis

For each presumptively positive sample, five plaques appearing on plates of the highest dilution were picked up for molecular identification. The templates used for PCR were individual plaques re-suspended in 100 ml of sterile double-distilled water and vortexed at a high speed. The liquid phase was transferred to a new tube and subjected to heating in boiling water for 3 min (Jurkevitch et al., 2000). 16S rRNA PCR analysis was performed on a fragment of the 16S rRNA gene of the *Halobacteriovoraceae* family members using the primers Bac676F and Bac1442R, as previously described (Davidov et al., 2006; Richards et al., 2013). Samples showing a band of 700 bp were considered *Halobacteriovorax*. For each positive sample, sequencing analysis was performed on PCR product of 700 bp from a unique plaque. PCR products were purified with the High Pure PCR Product Purification kit Roche Diagnostics (GmbH, Mannheim, Germany). Sequencing analysis was performed using the reverse primer BAC1442R and ABI Prism^®^ BigDye^®^ Terminator v1.1 Cycle Sequencing kit (Applied Biosystems^TM^, Life Technologies, United States), according to the manufacturer’s instructions. Sequenced products were analyzed in an automated capillary sequencer ABI Prism^®^ 310 Genetic Analyzer (Applied Biosystems^TM^, United States). Nucleotide sequences were manually edited, aligned and analyzed using CLC genomics workbench V.12 (Qiagen Bioinformatics). Phylogenetic trees of the 16S rRNA gene sequences from the isolates generated in this study were reconstructed using the maximum likelihood (ML) method. These sequences were also compared to those of the following type strains of the *Halobacteriovorax* species: *H. marinus* SJ (GenBank accession number 102485), *H. litoralis* JS5 (GenBank accession number 028724) and *H. vibrionivorans* BL9 (GenBank accession number MH150810) Initially the 16SrRNA sequences were aligned with a progressive alignment tool (Feng and Doolittle, 1987) within the CLC genomics workbench 20 environment. The best substitution model fitting the alignment was the General Time Reversible (GTR), gamma distribution 0 and transition/transversion rate ratio 2. The tree topology was tested with 1000 bootstrap replicates.

### Total *Vibrio* spp. and *V. parahaemolyticus* Enumeration

Each water sample was mixed and 100 ml of undiluted and 1:10, 1:100, 1:1000 diluted sample was filtered using a 0.45-mm-pore membrane filter (Millipore, Bedford, MA, United States); the filter was placed on thiosulfatecitrate-bile-salts-sucrose-agar (TCBS, Difco Laboratories, Detroit, MI, United States) and incubated at 37°C for 24 h. For each presumptively positive sample, five colonies appearing on plates of the highest dilution were selected and subcultured on trypticase soy agar with 2% NaCl (TSAs, Oxoid). Presumptive *Vibrio* spp. were biochemically identified at genus level by a standardized protocol (Ottaviani et al., 2003). Presumptive *V. parahaemolyticus* strains were confirmed by a standard method (ISO/TS, 2017). The number of viable *V. parahaemolyticus* and total *Vibrio* isolates was estimated as CFU per 100 ml seawater.

### Prey Specificity and Predatory Efficiency of *Halobacteriovorax* Isolates

Three-day enrichments of each *Halobacteriovorax* isolate (approximately 1 × 10^6^ PFU ml^–1^) were filtered through a 0.45- m-pore-size Millex HV syringe filter (Millipore Corp., Billerica, MA, United States) to remove the primary prey. Prey specificity and predator efficiency of *Halobacteriovorax* strains were determined by monitoring their abilities to form clear lytic halos on a lawn of the reference *V. parahaemolyticus* and *V. cholerae* preys listed in Table 1, using a double layer agar plating technique. Briefly, 1 μl of filtered predator undiluted enrichment, 1 ml of prey culture at an OD_600_ of 0.2 for *Vibrio* preys (approximately 10^8^ PFU ml^–1^), were added to 7.5 ml of sterilized artificial seawater (30 ppt salinity) and 7.5 ml of Pp 20 top agar (Richards et al., 2012, 2016). For each prey specificity assay a positive control, represented by *V. parahaemolyticus* NCTC 10885 and the *Halobacteriovorax* strain, and a negative control, which consisted of the prey alone, were performed.

### Statistical Analysis

On each 2 l seawater sample the analyses were performed in six independent replicates for *Halobacteriovorax* and *Vibrio* (*n* = 6). Results of microbiological analyses were reported as mean values (log-transformed) ± standard deviation. Correlation coefficients were determined and associated *p*-values < 0.05 were considered significant.

## Results

### *Halobacteriovorax* Enumeration

**FIGURE 2:**
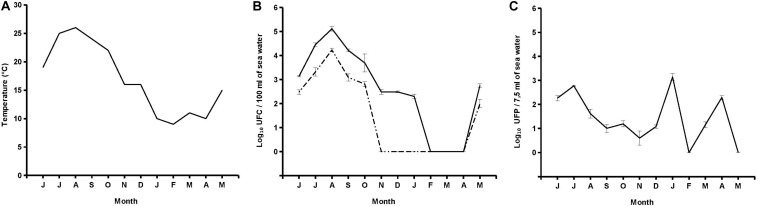
Trend of temperature **(A)**, *V. parahaemolyticus* and total *vibrio* **(B)**, and *Halobacteriovorax* **(C)**.

Out of 12 seawater samples collected, 10 (83.3%) were positive for presumptive *V. parahaemolyticus* specific *Halobacteriovorax*, with counts ranging from 4 PFU per 7.5 ml (November 2017) to 1.4 × 10^3^ PFU per 7.5 ml (January 2018) (Figure 2). Plaques on primary prey became visible after 72 h. After that, sizes expanded over time, reaching the maximum diameter of 9 mm after 5 days of incubation at 26°C. A lack of plaques was observed on all replicates of February and May 2018 samples even when the incubation was prolonged up to 10 days. No significant relationship was found between *Halobacteriovorax* levels and seawater temperature (Figure 2).

### *Halobacteriovorax* Molecular Identification and Sequencing Analysis

**FIGURE 3:**
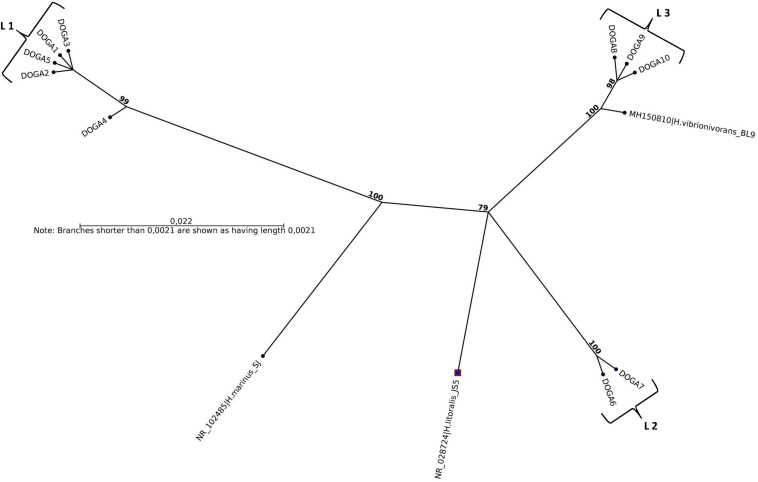
Maximum-likelihood phylogenetic tree, based on 16S rRNA gene sequence comparisons, showing the position of strains DOGA1-10 and related type strains. Numbers at branch nodes are bootstrap values (per 1000 trials).

All plaques for each seawater sample were confirmed by molecular methods as *Halobacteriovorax.* The sequences obtained by analysis of the 10 16SrRNA 700pb PCR fragments, one for each positive sample, were named DOGA1-DOGA10. The partial 16SrRNA gene sequences were deposited in GenBank under accession numbers MN750616–MN750625 (Table 2). Sequences from DOGA1 to DOGA7 were obtained from the strains isolated in 2017 while sequences from DOGA8 to DOGA10 were from the strains isolated in 2018 (Table 2). Overall, the divergence between our sequences was always <10% (Supplementary Figure 1). A similarity >99% was between the sequences DOGA1-DOGA5, DOGA6-DOGA7 and DOGA8-DOGA10. Between the three groups, the sequence similarity ranged from 90.63% (between DOGA 1 and DOGA 8) to 94.51% (between DOGA 6 and DOGA 9–10) (Supplementary Figure 1). These three distinct lineages were named L1, L2, L3, respectively (Table 2). DOGA2, DOGA3, and DOGA5 within L1 showed 100% sequence identity (Figure 3 and Table 2). Also DOGA9 and DOGA10 within L3 showed 100% sequence identity (Figure 3 and Table 2). Sequences within L3 shared 98.54–99.19% similarity with *H. vibrionivorans* BL9, 94.83–95.32% with *H. litoralis* JS5 and 91.75–92.23%. with *H. marinus* SJ (Figure 3 and Supplementary Figure 1). Sequences within L2 shared 94.18–94.51% similarity with *H. vibrionivorans* BL9, 95.64–95.96% with *H. litoralis* JS5 and 93.38–93.70%. with *H. marinus* SJ (Figure 3 and Supplementary Figure 1). Sequences within L1 shared 91.44–92.57% similarity with *H. vibrionivorans* BL9, 91.11–92.25% with *H. litoralis* JS5 and 92.41–93.21%. with *H. marinus* SJ (Figure 3 and Supplementary Figure 1).

**TABLE 2:** *Halobacteriovorax* strains molecularly characterized in this study.

16S-rRNA sequences	GenBank accession number	Period of isolation	Seawater temperature	16S-rRNA lineages
DOGA1	MN750616	June 2017	20°C	L1
DOGA2	MN750617	July 2017	25°C	L1
DOGA3	MN750618	August 2017	26°C	L1
DOGA4	MN750619	September 2017	24°C	L1
DOGA5	MN750620	October 2017	22°C	L1
DOGA6	MN750621	November 2017	16°C	L2
DOGA7	MN750622	December 2017	16°C	L2
DOGA8	MN750623	January 2018	10°C	L3
DOGA9	MN750624	March 2018	11°C	L3
DOGA10	MN750625	April 2018	10°C	L3

### Total *Vibrio* spp. and *V. parahaemolyticus* Enumeration

Six samples (50%) were positive for *V. parahaemolyticus* with counts ranging from 1.0 × 10^2^ CFU per 100 ml (May 2018) to 1.7 × 10^4^ CFU per 100 ml (August 2017). A lack of detection was reported from November to December 2017 and from January to April 2018. Nine samples (75%) were positive for total vibrios with counts ranging from 2.0 × 10^2^ CFU per 100 ml (January 2018) to 1.3 × 10^5^ CFU per 100 ml (August 2017). A lack of detection was reported from February to April 2018. No significant relationship was found between levels of *Halobacteriovorax* and those of total *Vibrio* and *V. parahaemolyticus* (Figure 2).

### Prey Specificity and Predatory Efficiency of *Halobacteriovorax* Isolates

All *Halobacteriovorax* isolates were able to lyse all tested *V. parahaemolyticus* and *V. cholerae* reference strains. Plaques of lysis became visible after 72 h and then, their sizes expanded over time, reaching the maximum after 5 days of incubation at 26°C. At the end of the incubation, the diameter of lysis plaques on vibrios was similar to those on primary prey, ranging between 7 and 9 mm diameter.

## Discussion

### *Halobacteriovorax* Enumeration

Indigenous *V. parahaemolyticus*-specific *Halobacteriovorax* is present in seawater of a mussel growing area of the Conero Riviera at levels higher than those reported for Atlantic and Pacific Oceans which never exceeded values of 10^2^ PFU per mL (Richards et al., 2013). Moreover, the *Halobacteriovorax* counts in this study should be considered minimum counts since some portion of the predators were undoubtedly removed by filtration (Richards et al., 2013). Surprisingly, we found the *Halobacteriovorax* higher abundances on January 2018 while *Halobacteriovorax* in Atlantic and Pacific Oceans were mainly concentrated in the warmer months of the year (Richards et al., 2013). It is known that *Halobacteriovorax* against *V. parahaemolyticus* is able to proliferate in the temperature range between 10 and 30°C (Williams et al., 2015). In the marine areas investigated by Richards et al. (2013) temperatures over a 12-month period ranged from lows of 5°C in winter and highs of 30°C in summer. *Halobacteriovorax* replication was therefore completely inhibited in the winter months. In the Conero Riviera temperatures over a 12-month period ranged from lows of 10°C in winter and highs of 25°C in summer. Therefore, throughout the year, the temperatures were suitable for a good replication of *Halobacteriovorax*.

### *Halobacteriovorax* Molecular Identification and Sequencing Analysis

In BALOs, as in other bacteria, a 90% sequence similarity means grouping at the genus level while the species level is identified by a >98% similarity (Koval et al., 2015). In this study the sequence similarity of *Halobacteriovorax* strains inter-lineages was always <94.5% suggesting that they may therefore well belong to three different species. Moreover, sequences in L1 and L2 formed independent branches separated from the type strains of *Halobacteriovorax.* Instead the sequences in L3 shared >99% similarity with *H. vibrionivorans* BL9. For this reason these *Halobacteriovorax* strains would clustered with the proposed new species *H. vibrionivorans.* In this period there is a great interest from the scientific world in the taxonomic and phylogenetic study of this genus. Shortly we will perform the genome sequencing of our *Halobacteriovorax* strains in order to obtain more detailed information on their taxonomic position and understand if they could belong to new species within the genus. Our results show that different strains of *Halobacteriovorax* circulated in the Conero Riviera in the 2017–2018 period. In this study we have limited ourselves to the molecular characterization of the dominant population of *Halobacteriovorax*. For this reason, the plaques for each isolate were enumerated and treated as if they were a single clonal population. Moreover, in this study we have used a unique *V. parahaemolyticus* strain as prey to recover *Halobacteriovorax*. These working methods may have limited the *Halobacteriovorax* diversity detected in the marine area investigated. Previous studies associated different clusters of *Halobacteriovorax* to marine waters with different salinity (Pineiro et al., 2007, 2013; Crossman et al., 2013; Richards et al., 2013). In disagreement, our strains belonging to different lineages, were isolated from a marine area with a constant level of salinity equal to 38–39 ppt throughout the year. An association was found among the lineages of our *Halobacteriovorax* strains and the temperature of seawater. The strains in L1 were isolated in the period June–October 2017 when the temperature ranged between 20 and 25°C. The strains in L2 were isolated in the period November–December 2017 when the temperature was 16°C. Finally, the strains in L3 were isolated in the period January–April 2018 when the temperature ranged between 10 and 11°C. In a future investigation we will extend the period and marine area of the study, by using different *Vibrio parahemolyticus* prey strains and sequencing 16SrRNA fragments from more plaques for each sample. The scope will be to assess if, in the Adriatic Sea, diversities of *Halobacteriovorax* may exist in respect of seasonality and prey specificity and whether particular strains can be present here stably.

### Total *Vibrio* spp. and *V. parahaemolyticus* Enumeration

No significant correlations between *Halobacteriovorax*, total *Vibrio* spp. and *V. parahaemolyticus* abundance was detected in the marine area investigated in this study. A previous study confined to microcosms in which *Halobacteriovorax*/*V. parahaemolyticus* at a ratio of 10^5^ PFU/10^5^ CFU per ml were added, produced a different effect, where *Halobacteriovorax* increased as *V. parahaemolyticus* decreased (Ottaviani et al., 2018a). The fact that no relationship between the abundance of *Halobacteriovorax* and *V. parahaemolyticus* was observed in the present study could be related to the very low abundance of both genera in seawater which was about 10–100 cells per ml. This may have reduced the chance for high predation and consequently a significant increase and decrease in the abundance of *Halobacteriovorax* and *V. parahaemolyticus*, respectively. Moreover, it is known that some *Halobacteriovorax* has broader host specificity than others and the mechanisms that drive *Halobacteriovorax* host specificity within a complex bacterial community are not known. In this regard, our *Halobacteriovorax* strains demonstrated predatory activity *in vitro* toward all *V. parahaemolyticus* and *V. cholerae* reference strains tested, many of them isolated from the same marine area. *Halobacteriovorax* may well target one microorganism over another within the same species and also between different species of marine bacteria. The extent to which this occurs remains unresolved. In the next future it would be essential to perform microcosm-studies to investigate the unwanted effect of *Halobacteriovorax* isolates toward the vital core-microbiome bacteria in marine seawater. Moreover, *Halobacteriovorax* and *Vibrio* abundances may be subject to control by bacteriophages, other predators, environmental conditions, nutrient levels, the effects of competing microbes, and the development of host resistance. Finally, the lack of correlation between predator and prey levels could be linked to the different speed of *Halobacteriovorax* and *Vibrio* replication, so that they cannot compete with maximum efficiency between them.

### Prey Specificity and Predatory Efficiency of *Halobacteriovorax* Isolates

Our *Halobacteriovorax* isolates, although captured using a single *V. parahaemolyticus* strain as primary prey, were able to lyse a wide range of *V. parahaemolyticus* and *V. cholerae* strains, including those toxigenic and/or involved in human infections. Previous scientific information reported that *V. parahaemolyticus* is the most efficient prey known for *Halobacteriovorax* recovery, at least for the strains against *Vibrio* (Pineiro et al., 2007). The broad spectrum of action of our isolates would seem to confirm this hypothesis. Most of the vibrio prey came from the same marine area from which *Halobacteriovorax* strains were also isolated. Some had been isolated from mussels, others from seawater in the Conero Riviera. We believe that *Halobacteriovorax* similarly to other bacteria through the filtration from seawater passes inside the mollusk where continues to parasitize vibrios and other potential pathogens. A previous study investigated the *Halobacteriovorax* predation in coral-associated microbiome (Welsh et al., 2016). The authors speculated that coral microbiome could allow the predator to remain alive and vital while *Halobacteriovorax* could regulate and maintain the microbiome structure in balance and at the same time protect the host by consuming potential pathogens. Similar symbiotic relationships may also exist between *Halobacteriovorax* and the microbiome associated with other aquatic organisms, such as bivalves. Laboratory-scale studies are currently underway to evaluate the effects of *Halobacteriovorax* in the reduction of *V. parahaemolyticus* in bivalves during the purification phase and to understand the potential unwanted effects of the predator on the mussel microbiome.

## Conclusion

In conclusion, different *V. parahaemolyticus*-specific *Halobacteriovorax* strains are present in seawater of a mussel growing area on the Conero Riviera where they, probably, play a physiological role as natural modulators versus *V. parahaemolyticus* and other vibrios populations. These predatory bacteria could have a primary role in regulating and structuring marine bacterial communities and the nutrient cycle but have not received the attention they deserve to date. Additional research is necessary to better understand the mechanisms by which *Halobacteriovorax* may modulate *V. parahaemolyticus* and other vibrios in a complex marine ecosystem, and the overall effect they exert on the structure of seawater microbial communities and marine host-associated microbiomes.

## Data Availability Statement

The datasets generated for this study can be found in the GenBank under the following accession numbers: SUB6636935 DOGA1 MN750616, SUB6636935 DOGA2 MN750617, SUB6636935 DOGA3 MN750618, SUB6636935 DOGA4 MN750619, SUB6636935 DOGA5 MN750620, SUB6636935 DOGA6 MN750621, SUB6636935 DOGA7 MN750622, SUB6636935 DOGA8 MN750623, SUB6636935 DOGA9 MN750624, and SUB6636935 DOGA10 MN750625.

## Author Contributions

GA, SP, and FM isolated bacteria and performed the laboratory measurements. DO, FL, ER, ML, and PT made substantial contributions to conception and design. AP aligned and compared 16SrRNA sequences. DO wrote and revised the manuscript. DO and GA drafted the manuscript. All authors contributed to the article and approved the submitted version.

## Conflict of Interest

The authors declare that the research was conducted in the absence of any commercial or financial relationships that could be construed as a potential conflict of interest.
