# Supplementary figures and images for: Vibrio parahaemolyticus-specific Halobacteriovorax From Seawater of a Mussel Harvesting Area in the Adriatic Sea: Abundance, Diversity, Efficiency and Relationship With the Prey Natural Level
Source: Front Microbiol. 2020 Jul 8;11:1575. doi: 10.3389/fmicb.2020.01575 (PMC7360731; doi:10.3389/fmicb.2020.01575)

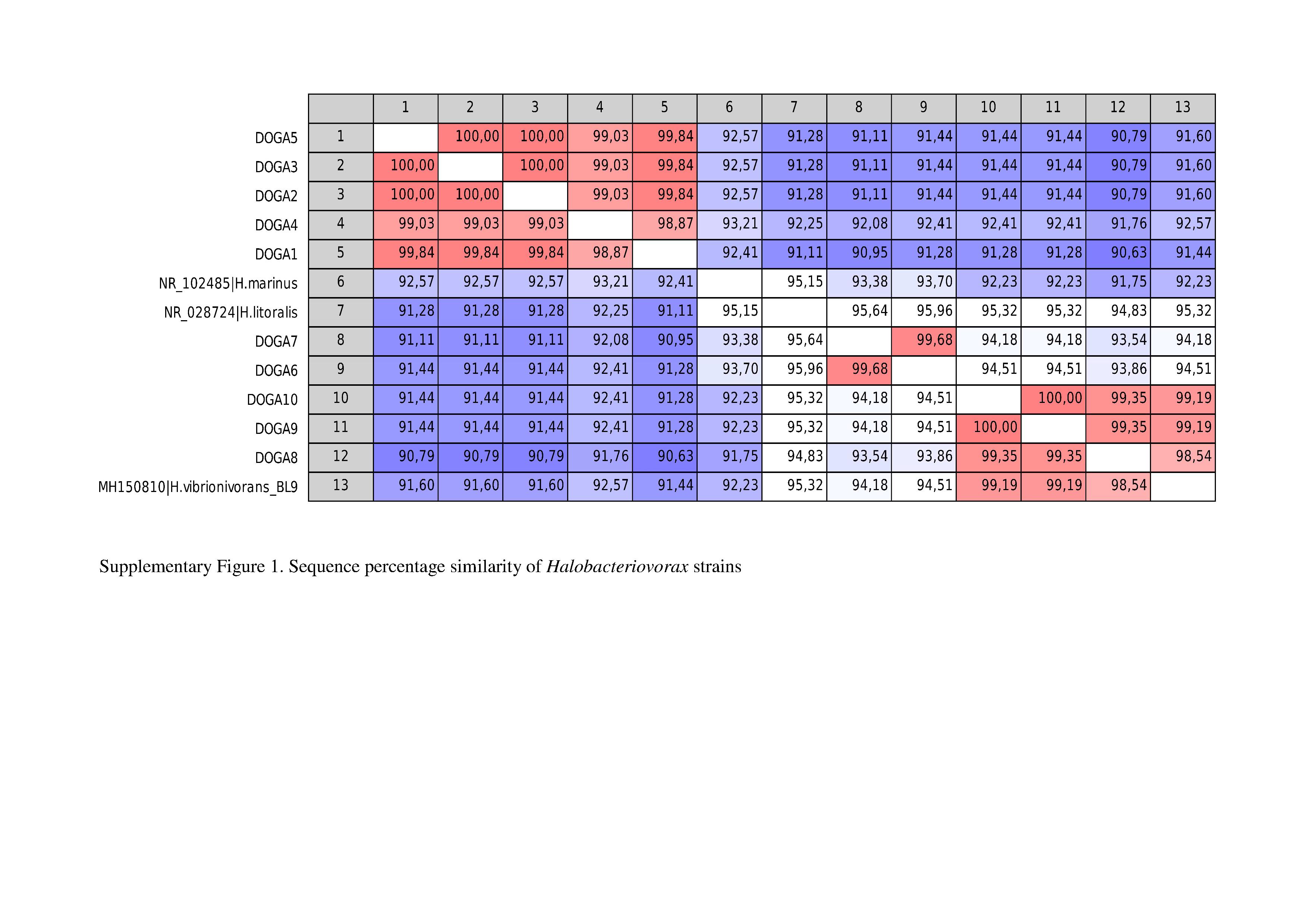

Supplement: Supplementary file 1 [file Image_1.jpg]
